# Anxiety, Affect, Self-Esteem, and Stress: Mediation and Moderation Effects on Depression

**DOI:** 10.1371/journal.pone.0073265

**Published:** 2013-09-09

**Authors:** Ali Al Nima, Patricia Rosenberg, Trevor Archer, Danilo Garcia

**Affiliations:** 1 Department of Psychology, University of Gothenburg, Gothenburg, Sweden; 2 Network for Empowerment and Well-Being, University of Gothenburg, Gothenburg, Sweden; 3 Department of Psychology, Education and Sport Science, Linneaus University, Kalmar, Sweden; 4 Center for Ethics, Law, and Mental Health (CELAM), University of Gothenburg, Gothenburg, Sweden; 5 Institute of Neuroscience and Physiology, The Sahlgrenska Academy, University of Gothenburg, Gothenburg, Sweden; The University of Melbourne, Australia

## Abstract

**Background:**

Mediation analysis investigates whether a variable (i.e., mediator) changes in regard to an independent variable, in turn, affecting a dependent variable. Moderation analysis, on the other hand, investigates whether the statistical interaction between independent variables predict a dependent variable. Although this difference between these two types of analysis is explicit in current literature, there is still confusion with regard to the mediating and moderating effects of different variables on depression. The purpose of this study was to assess the mediating and moderating effects of anxiety, stress, positive affect, and negative affect on depression.

**Methods:**

Two hundred and two university students (males  = 93, females  = 113) completed questionnaires assessing anxiety, stress, self-esteem, positive and negative affect, and depression. Mediation and moderation analyses were conducted using techniques based on standard multiple regression and hierarchical regression analyses.

**Main Findings:**

The results indicated that (i) anxiety partially mediated the effects of both stress and self-esteem upon depression, (ii) that stress partially mediated the effects of anxiety and positive affect upon depression, (iii) that stress completely mediated the effects of self-esteem on depression, and (iv) that there was a significant interaction between stress and negative affect, and between positive affect and negative affect upon depression.

**Conclusion:**

The study highlights different research questions that can be investigated depending on whether researchers decide to use the same variables as mediators and/or moderators.

## Introduction

Mediation refers to the covariance relationships among three variables: an independent variable (1), an assumed mediating variable (2), and a dependent variable (3). Mediation analysis investigates whether the mediating variable accounts for a significant amount of the shared variance between the independent and the dependent variables–the mediator changes in regard to the independent variable, in turn, affecting the dependent one [1], [2]. On the other hand, moderation refers to the examination of the statistical interaction between independent variables in predicting a dependent variable [1], [3]. In contrast to the mediator, the moderator is not expected to be correlated with both the independent and the dependent variable–Baron and Kenny [1] actually recommend that it is best if the moderator is not correlated with the independent variable and if the moderator is relatively stable, like a demographic variable (e.g., gender, socio-economic status) or a personality trait (e.g., affectivity).

Although both types of analysis lead to different conclusions [3] and the distinction between statistical procedures is part of the current literature [2], there is still confusion about the use of moderation and mediation analyses using data pertaining to the prediction of depression. There are, for example, contradictions among studies that investigate mediating and moderating effects of anxiety, stress, self-esteem, and affect on depression. Depression, anxiety and stress are suggested to influence individuals' social relations and activities, work, and studies, as well as compromising decision-making and coping strategies [4], [5], [6]. Successfully coping with anxiety, depressiveness, and stressful situations may contribute to high levels of self-esteem and self-confidence, in addition increasing well-being, and psychological and physical health [6]. Thus, it is important to disentangle how these variables are related to each other. However, while some researchers perform mediation analysis with some of the variables mentioned here, other researchers conduct moderation analysis with the same variables. Seldom are both moderation and mediation performed on the same dataset. Before disentangling mediation and moderation effects on depression in the current literature, we briefly present the methodology behind the analysis performed in this study.

### Mediation and moderation

Baron and Kenny [1] postulated several criteria for the analysis of a mediating effect: a significant correlation between the independent and the dependent variable, the independent variable must be significantly associated with the mediator, the mediator predicts the dependent variable even when the independent variable is controlled for, and the correlation between the independent and the dependent variable must be eliminated or reduced when the mediator is controlled for. All the criteria is then tested using the Sobel test which shows whether indirect effects are significant or not [1], [7]. A complete mediating effect occurs when the correlation between the independent and the dependent variable are eliminated when the mediator is controlled for [8]. Analyses of mediation can, for example, help researchers to move beyond answering if high levels of stress lead to high levels of depression. With mediation analysis researchers might instead answer *how* stress is related to depression.

In contrast to mediation, moderation investigates the unique conditions under which two variables are related [3]. The third variable here, the moderator, is not an intermediate variable in the causal sequence from the independent to the dependent variable. For the analysis of moderation effects, the relation between the independent and dependent variable must be different at different levels of the moderator [3]. Moderators are included in the statistical analysis as an interaction term [1]. When analyzing moderating effects the variables should first be centered (i.e., calculating the *mean* to become 0 and the standard deviation to become 1) in order to avoid problems with multi-colinearity [8]. Moderating effects can be calculated using multiple hierarchical linear regressions whereby main effects are presented in the first step and interactions in the second step [1]. Analysis of moderation, for example, helps researchers to answer *when* or *under which conditions* stress is related to depression.

### Mediation and moderation effects on depression

Cognitive vulnerability models suggest that maladaptive self-schema mirroring helplessness and low self-esteem explain the development and maintenance of depression (for a review see [9]). These cognitive vulnerability factors become activated by negative life events or negative moods [10] and are suggested to interact with environmental stressors to increase risk for depression and other emotional disorders [11], [10]. In this line of thinking, the experience of stress, low self-esteem, and negative emotions can cause depression, but also be used to explain *how* (i.e., mediation) and *under which conditions* (i.e., moderation) specific variables influence depression.

Using mediational analyses to investigate how cognitive therapy intervations reduced depression, researchers have showed that the intervention reduced anxiety, which in turn was responsible for 91% of the reduction in depression [12]. In the same study, reductions in depression, by the intervention, accounted only for 6% of the reduction in anxiety. Thus, anxiety seems to affect depression more than depression affects anxiety and, together with stress, is both a cause of and a powerful mediator influencing depression (See also [13]). Indeed, there are positive relationships between depression, anxiety and stress in different cultures [14]. Moreover, while some studies show that stress (independent variable) increases anxiety (mediator), which in turn increased depression (dependent variable) [14], other studies show that stress (moderator) interacts with maladaptive self-schemata (dependent variable) to increase depression (independent variable) [15], [16].

### The present study

In order to illustrate how mediation and moderation can be used to address different research questions we first focus our attention to anxiety and stress as mediators of different variables that earlier have been shown to be related to depression. Secondly, we use all variables to find which of these variables moderate the effects on depression.

The specific aims of the present study were:

1. To investigate if anxiety mediated the effect of stress, self-esteem, and affect on depression.
2. To investigate if stress mediated the effects of anxiety, self-esteem, and affect on depression.
3. To examine moderation effects between anxiety, stress, self-esteem, and affect on depression.

## Methods

### Ethics statement

This research protocol was approved by the Ethics Committee of the University of Gothenburg and written informed consent was obtained from all the study participants.

### Participants

The present study was based upon a sample of 206 participants (males  = 93, females  = 113). All the participants were first year students in different disciplines at two universities in South Sweden. The mean age for the male students was 25.93 years (*SD* = 6.66), and 25.30 years (*SD* = 5.83) for the female students.

In total, 206 questionnaires were distributed to the students. Together 202 questionnaires were responded to leaving a total dropout of 1.94%. This dropout concerned three sections that the participants chose not to respond to at all, and one section that was completed incorrectly. None of these four questionnaires was included in the analyses.

### Instruments

#### Hospital Anxiety and Depression Scale [17]

The Swedish translation of this instrument [18] was used to measure anxiety and depression. The instrument consists of 14 statements (7 of which measure depression and 7 measure anxiety) to which participants are asked to respond grade of agreement on a Likert scale (0 to 3). The utility, reliability and validity of the instrument has been shown in multiple studies (e.g., [19]).

#### Perceived Stress Scale [20]

The Swedish version [21] of this instrument was used to measures individuals' experience of stress. The instrument consist of 14 statements to which participants rate on a Likert scale (0 =  *never*, 4 =  *very often*). High values indicate that the individual expresses a high degree of stress.

#### Rosenberg's Self-Esteem Scale [22]

The Rosenberg's Self-Esteem Scale (Swedish version by Lindwall [23]) consists of 10 statements focusing on general feelings toward the self. Participants are asked to report grade of agreement in a four-point Likert scale (1 =  *agree not at all,* 4 =  *agree completely*). This is the most widely used instrument for estimation of self-esteem with high levels of reliability and validity (e.g., [24], [25]).

#### Positive Affect and Negative Affect Schedule [26]

This is a widely applied instrument for measuring individuals' self-reported mood and feelings. The Swedish version has been used among participants of different ages and occupations (e.g., [27], [28], [29]). The instrument consists of 20 adjectives, 10 positive affect (e.g., proud, strong) and 10 negative affect (e.g., afraid, irritable). The adjectives are rated on a five-point Likert scale (1 =  *not at all*, 5 =  *very much*). The instrument is a reliable, valid, and effective self-report instrument for estimating these two important and independent aspects of mood [26].

### Procedure

Questionnaires were distributed to the participants on several different locations within the university, including the library and lecture halls. Participants were asked to complete the questionnaire after being informed about the purpose and duration (10–15 minutes) of the study. Participants were also ensured complete anonymity and informed that they could end their participation whenever they liked.

## Results

### Correlational analysis

Depression showed positive, significant relationships with anxiety, stress and negative affect. Table 1 presents the correlation coefficients, *mean* values and *standard deviations* (*sd*), as well as *Cronbach*'*s α* for all the variables in the study.

**Table 1:** Correlations, means, standard deviations and Cronbach's α for all the variables in the study.

	1	2	3	4	5	6	7	8
(1) Gender	−							
(2) Age	−.05	−						
(3) Depression	.08	−.01	−					
(4) Anxiety	.21**	−.06	.63**	−				
(5) Stress	.22**	−.18**	.64**	.72**	−			
(6) Self-esteem	−.24**	.09	−60**	−.70**	−.66**	−		
(7) Positive Affect	−.14*	.13	−.54**	−.46**	−.58**	.54**	−	
(8) Negative Affect	.21**	−.21**	.50**	.69**	.69**	−.63**	−.37**	−
Mean and Sd.	−	25.58±6.20	3.69±2.75	7.67±3.93	26.04±8.74	28.51±5.59	34.71±7.08	22.10±7.59
*Cronbach*'*s α*	−	−	.70	.82	.85	.84	.87	.85

Note: * *p*<.05, ** *p*<.01.

### Mediation analysis

Regression analyses were performed in order to investigate if anxiety mediated the effect of stress, self-esteem, and affect on depression (aim 1). The first regression showed that stress (*B* = .03, 95% CI [.02,.05], β = .36, *t* = 4.32, *p*<.001), self-esteem (*B* = −.03, 95% CI [−.05, −.01], β = −.24, *t* = −3.20, *p*<.001), and positive affect (*B* = −.02, 95% CI [−.05, −.01], β = −.19, *t* = −2.93, *p* = .004) had each an unique effect on depression. Surprisingly, negative affect did not predict depression (*p* = 0.77) and was therefore removed from the mediation model, thus not included in further analysis.

The second regression tested whether stress, self-esteem and positive affect uniquely predicted the mediator (i.e., anxiety). Stress was found to be positively associated (*B* = .21, 95% CI [.15,.27], β = .47, *t* = 7.35, *p*<.001), whereas self-esteem was negatively associated (*B* = −.29, 95% CI [−.38, −.21], β = −.42, *t* = −6.48, *p*<.001) to anxiety. Positive affect, however, was not associated to anxiety (*p* = .50) and was therefore removed from further analysis.

A hierarchical regression analysis using depression as the outcome variable was performed using stress and self-esteem as predictors in the first step, and anxiety as predictor in the second step. This analysis allows the examination of whether stress and self-esteem predict depression and if this relation is weaken in the presence of anxiety as the mediator. The result indicated that, in the first step, both stress (*B* = .04, 95% CI [.03,.05], β = .45, *t* = 6.43, *p*<.001) and self-esteem (*B* = .04, 95% CI [.03,.05], β = .45, *t* = 6.43, *p*<.001) predicted depression. When anxiety (i.e., the mediator) was controlled for predictability was reduced somewhat but was still significant for stress (*B* = .03, 95% CI [.02,.04], β = .33, *t* = 4.29, *p*<.001) and for self-esteem (*B* = −.03, 95% CI [−.05, −.01], β = −.20, *t* = −2.62, *p* = .009). Anxiety, as a mediator, predicted depression even when both stress and self-esteem were controlled for (*B* = .05, 95% CI [.02,.08], β = .26, *t* = 3.17, *p* = .002). Anxiety improved the prediction of depression over-and-above the independent variables (i.e., stress and self-esteem) (Δ*R* ^2^ = .03, *F* (1, 198) = 10.06, *p* = .002). See Table 2 for the details.

**Table 2:** Predictors for depression using anxiety as the mediator.

Predictor	Step 1	Step 2	
	*B*	*B*	95% CI
Stress	.04***	.03***	(.02,.04)
Self-esteem	−.04***	−.03**	(−.05, −.01)
Anxiety		.05**	(.02,.08)
*R^2^*	.47	.49	
*F*	86.61***	63.72***	
Δ R^2^		.03	
Δ *F*		10.06**	

Note: ** p<.01, *** p<.001.

**Figure 1:**
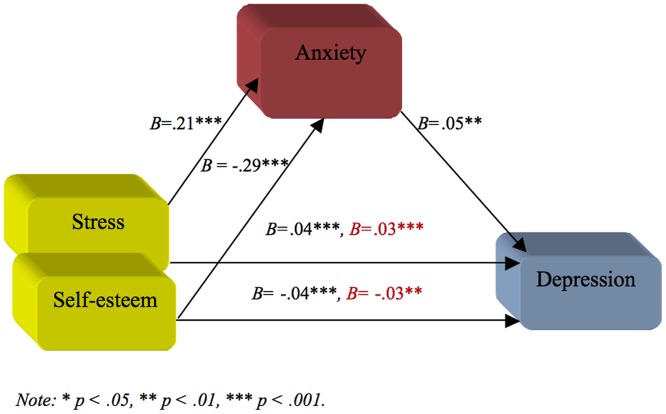
Mediation model showing that the effect of stress and self-esteem (independent variables) on depression (outcome) is mediated by anxiety (mediator). Changes in *Beta weights* when the mediator is present are highlighted in red.

A Sobel test was conducted to test the mediating criteria and to assess whether indirect effects were significant or not. The result showed that the complete pathway from stress (independent variable) to anxiety (mediator) to depression (dependent variable) was significant (*z* = 2.89, *p* = .003). The complete pathway from self-esteem (independent variable) to anxiety (mediator) to depression (dependent variable) was also significant (*z* = 2.82, *p* = .004). Thus, indicating that anxiety partially mediates the effects of both stress and self-esteem on depression. This result may indicate also that both stress and self-esteem contribute directly to explain the variation in depression and indirectly via experienced level of anxiety (see Figure 1).

For the second aim, regression analyses were performed in order to test if stress mediated the effect of anxiety, self-esteem, and affect on depression. The first regression showed that anxiety (*B* = .07, 95% CI [.04,.10], β = .37, *t* = 4.57, *p*<.001), self-esteem (*B* = −.02, 95% CI [−.05, −.01], β = −.18, *t* = −2.23, *p* = .03), and positive affect (*B* = −.03, 95% CI [−.04, −.02], β = −.27, *t* = −4.35, *p*<.001) predicted depression independently of each other. Negative affect did not predict depression (*p* = 0.74) and was therefore removed from further analysis.

The second regression investigated if anxiety, self-esteem and positive affect uniquely predicted the mediator (i.e., stress). Stress was positively associated to anxiety (*B* = 1.01, 95% CI [.75, 1.30], β = .46, *t* = 7.35, *p*<.001), negatively associated to self-esteem (*B* = −.30, 95% CI [−.50, −.01], β = −.19, *t* = −2.90, *p* = .004), and a negatively associated to positive affect (*B* = −.33, 95% CI [−.46, −.20], β = −.27, *t* = −5.02, *p*<.001).

A hierarchical regression analysis using depression as the outcome and anxiety, self-esteem, and positive affect as the predictors in the first step, and stress as the predictor in the second step, allowed the examination of whether anxiety, self-esteem and positive affect predicted depression and if this association would weaken when stress (i.e., the mediator) was present. In the first step of the regression anxiety (*B* = .07, 95% CI [.05,.10], β = .38, *t* = 5.31, *p* = .02), self-esteem (*B* = −.03, 95% CI [−.05, −.01], β = −.18, *t* = −2.41, *p* = .02), and positive affect (*B* = −.03, 95% CI [−.04, −.02], β = −.27, *t* = −4.36, *p*<.001) significantly explained depression. When stress (i.e., the mediator) was controlled for, predictability was reduced somewhat but was still significant for anxiety (*B* = .05, 95% CI [.02,.08], β = .05, *t* = 4.29, *p*<.001) and for positive affect (*B* = −.02, 95% CI [−.04, −.01], β = −.20, *t* = −3.16, *p* = .002), whereas self-esteem did not reach significance (*p*< = .08). In the second step, the mediator (i.e., stress) predicted depression even when anxiety, self-esteem, and positive affect were controlled for (*B* = .02, 95% CI [.08,.04], β = .25, *t* = 3.07, *p* = .002). Stress improved the prediction of depression over-and-above the independent variables (i.e., anxiety, self-esteem and positive affect) (Δ*R* ^2^ = .02, *F*(1, 197)  = 9.40, *p* = .002). See Table 3 for the details.

**Table 3:** Predictors for depression using stress as the mediator.

Predictor	Step 1	Step 2	
	*B*	*B*	95% CI
Anxiety	.07*	.05***	(.02,.08)
Self-esteem	−.03*	−.01	(−.04, −.01)
Positive Affect	−.03***	−.02**	
Stress		.02**	(.02,.08)
*R^2^*	.49	.52	
*F*	64.09***	52.46***	
Δ R^2^		.02	
Δ *F*		9.40**	

Note: * p<.05, ** p<.01, *** p<.001.

**Figure 2:**
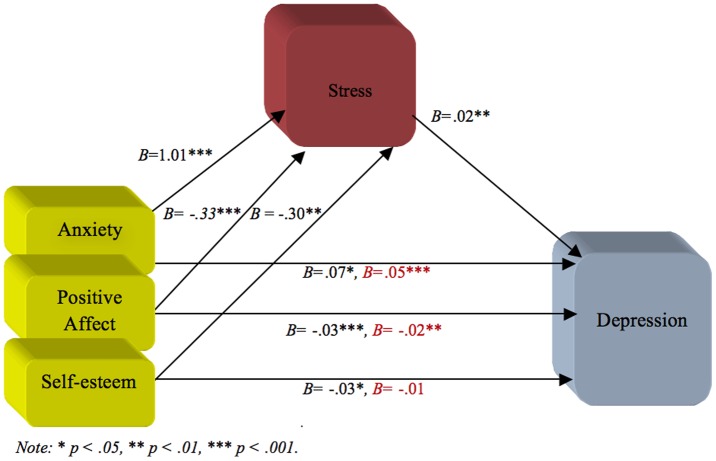
Mediation model showing that the effect of anxiety, positive affect, and self-esteem (dependent variables) on depression (outcome) is mediated by stress (mediator). Changes in *Beta weights* when the mediator is present are highlighted in red.

Furthermore, the Sobel test indicated that the complete pathways from the independent variables (anxiety: *z* = 2.81, *p* = .004; self-esteem: *z* =  2.05, *p* = .04; positive affect: *z* = 2.58, *p*<.01) to the mediator (i.e., stress), to the outcome (i.e., depression) were significant. These specific results might be explained on the basis that stress partially mediated the effects of both anxiety and positive affect on depression while stress completely mediated the effects of self-esteem on depression. In other words, anxiety and positive affect contributed directly to explain the variation in depression and indirectly via the experienced level of stress. Self-esteem contributed only indirectly via the experienced level of stress to explain the variation in depression. In other words, stress effects on depression originate from “its own power” and explained more of the variation in depression than self-esteem (see Figure 2).

### Moderation analysis

**Figure 3:**
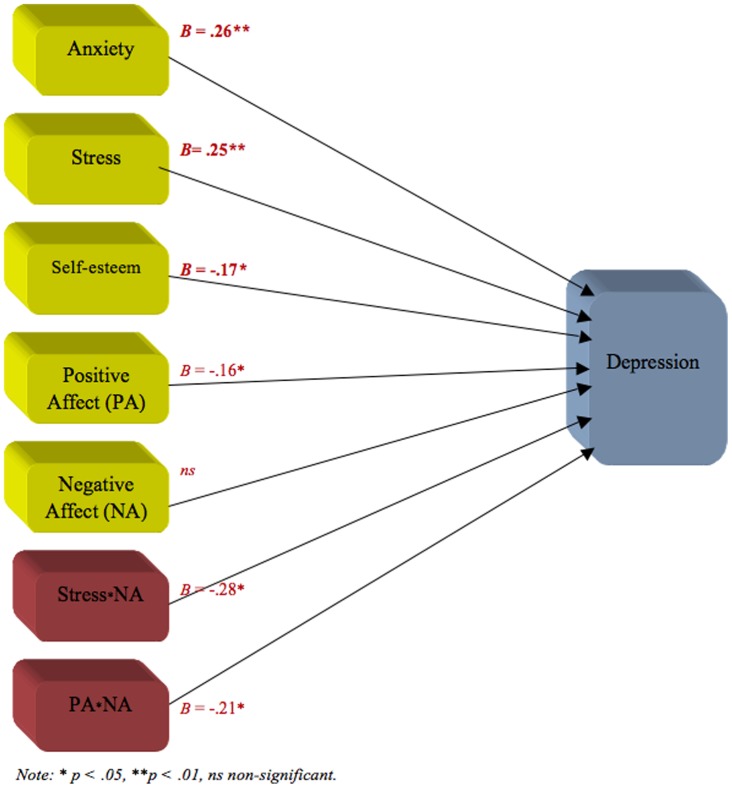
Moderation model showing all main effects and significant moderator effects on depression.

**Figure 4:**
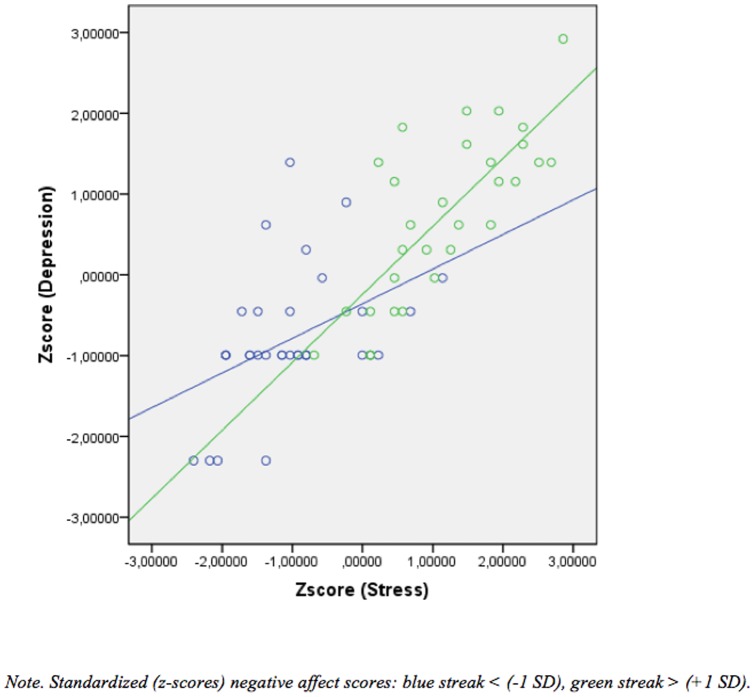
Showing the significant interaction between stress and negative affect upon depression. Low stress and low negative affect leads to lower levels of depression compared to high stress and high negative affect.

**Figure 5:**
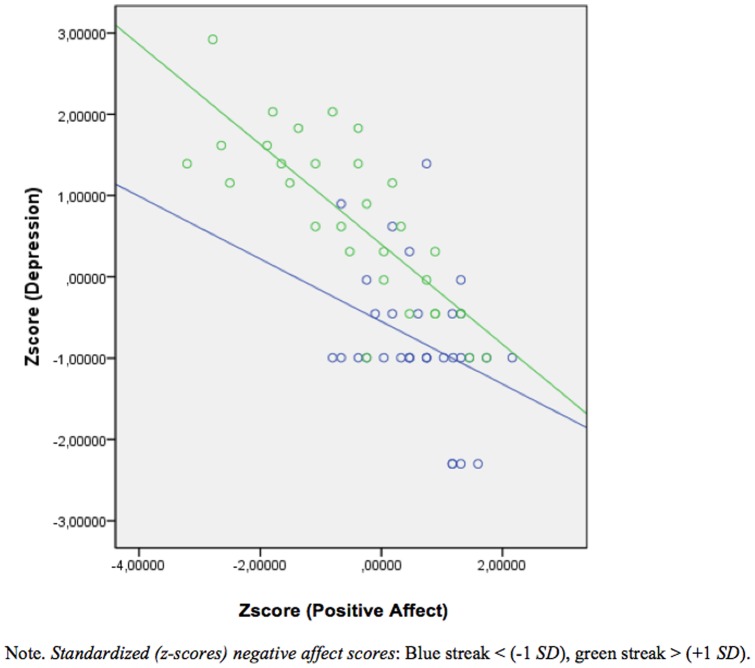
Showing the significant interaction between positive and negative affect on depression. High positive affect and low negative affect lead to lower levels of depression compared to low positive affect and high negative affect.

Multiple linear regression analyses were used in order to examine moderation effects between anxiety, stress, self-esteem and affect on depression. The analysis indicated that about 52% of the variation in the dependent variable (i.e., depression) could be explained by the main effects and the interaction effects (*R* ^2^ = .55, adjusted *R* ^2^ = .51, *F* (55, 186)  = 14.87, *p*<.001). When the variables (dependent and independent) were standardized, both the standardized regression coefficients beta (β) and the unstandardized regression coefficients beta (B) became the same value with regard to the main effects. Three of the main effects were significant and contributed uniquely to high levels of depression: anxiety (*B* = .26, *t* = 3.12, *p* = .002), stress (*B* = .25, *t* = 2.86, *p* = .005), and self-esteem (*B* = −.17, *t* = −2.17, *p* = .03). The main effect of positive affect was also significant and contributed to low levels of depression (*B* = −.16, *t* = −2.027, *p* = .02) (see Figure 3). Furthermore, the results indicated that two moderator effects were significant. These were the interaction between stress and negative affect (*B* = −.28, β = −.39, *t* = −2.36, *p* = .02) (see Figure 4) and the interaction between positive affect and negative affect (*B* = −.21, β = −.29, *t* = −2.30, *p* = .02) (Figure 5).

## Discussion

The results in the present study show that (i) anxiety partially mediated the effects of both stress and self-esteem on depression, (ii) that stress partially mediated the effects of anxiety and positive affect on depression, (iii) that stress completely mediated the effects of self-esteem on depression, and (iv) that there was a significant interaction between stress and negative affect, and positive affect and negative affect on depression.

### Mediating effects

The study suggests that anxiety contributes directly to explaining the variance in depression while stress and self-esteem might contribute directly to explaining the variance in depression and indirectly by increasing feelings of anxiety. Indeed, individuals who experience stress over a long period of time are susceptible to increased anxiety and depression [30], [31] and previous research shows that high self-esteem seems to buffer against anxiety and depression [32], [33]. The study also showed that stress partially mediated the effects of both anxiety and positive affect on depression and that stress completely mediated the effects of self-esteem on depression. Anxiety and positive affect contributed directly to explain the variation in depression and indirectly to the experienced level of stress. Self-esteem contributed only indirectly via the experienced level of stress to explain the variation in depression, i.e. stress affects depression on the basis of ‘its own power’ and explains much more of the variation in depressive experiences than self-esteem. In general, individuals who experience low anxiety and frequently experience positive affect seem to experience low stress, which might reduce their levels of depression. Academic stress, for instance, may increase the risk for experiencing depression among students [34]. Although self-esteem did not emerged as an important variable here, under circumstances in which difficulties in life become chronic, some researchers suggest that low self-esteem facilitates the experience of stress [35].

### Moderator effects/interaction effects

The present study showed that the interaction between stress and negative affect and between positive and negative affect influenced self-reported depression symptoms. Moderation effects between stress and negative affect imply that the students experiencing low levels of stress and low negative affect reported lower levels of depression than those who experience high levels of stress and high negative affect. This result confirms earlier findings that underline the strong positive association between negative affect and both stress and depression [36], [37]. Nevertheless, negative affect by itself did not predicted depression. In this regard, it is important to point out that the absence of positive emotions is a better predictor of morbidity than the presence of negative emotions [38], [39]. A modification to this statement, as illustrated by the results discussed next, could be that the presence of negative emotions in conjunction with the absence of positive emotions increases morbidity.

The moderating effects between positive and negative affect on the experience of depression imply that the students experiencing high levels of positive affect and low levels of negative affect reported lower levels of depression than those who experience low levels of positive affect and high levels of negative affect. This result fits previous observations indicating that different combinations of these affect dimensions are related to different measures of physical and mental health and well-being, such as, blood pressure, depression, quality of sleep, anxiety, life satisfaction, psychological well-being, and self-regulation [40]–[51].

### Limitations

The result indicated a relatively low mean value for depression (*M* = 3.69), perhaps because the studied population was university students. These might limit the generalization power of the results and might also explain why negative affect, commonly associated to depression, was not related to depression in the present study. Moreover, there is a potential influence of single source/single method variance on the findings, especially given the high correlation between all the variables under examination.

### Conclusions

The present study highlights different results that could be arrived depending on whether researchers decide to use variables as mediators or moderators. For example, when using meditational analyses, anxiety and stress seem to be important factors that explain how the different variables used here influence depression–increases in anxiety and stress by any other factor seem to lead to increases in depression. In contrast, when moderation analyses were used, the interaction of stress and affect predicted depression and the interaction of both affectivity dimensions (i.e., positive and negative affect) also predicted depression–stress might increase depression under the condition that the individual is high in negative affectivity, in turn, negative affectivity might increase depression under the condition that the individual experiences low positive affectivity.
