# Correction: Anxiety, Affect, Self-Esteem, and Stress: Mediation and Moderation Effects on Depression

**DOI:** 10.1371/annotation/49e2c5c8-e8a8-4011-80fc-02c6724b2acc

**Published:** 2013-09-23

**Authors:** Ali Al Nima, Patricia Rosenberg, Trevor Archer, Danilo Garcia

There was information omitted from the Funding statement. The correct version is available below.

This study was supported by Stiftelsen Kempe-Carlgrenska Fonden. The funders had no role in study design, data collection and analysis, decision to publish, or preparation of the manuscript. 

